# Spatial Patterns of Soil Respiration Links Above and Belowground Processes along a Boreal Aspen Fire Chronosequence

**DOI:** 10.1371/journal.pone.0165602

**Published:** 2016-11-10

**Authors:** Sanatan Das Gupta, M. Derek Mackenzie

**Affiliations:** 1 Natural Resources Canada, Canadian Forest Service, Northern Forestry Centre, Edmonton, AB, Canada; 2 Department of Renewable Resources, University of Alberta, Edmonton AB, Canada; The University of Auckland, NEW ZEALAND

## Abstract

Fire in boreal ecosystems is known to affect CO_2_ efflux from forest soils, which is commonly termed soil respiration (Rs). However, there is limited information on how fire and recovery from this disturbance affects spatial variation in Rs. The main objective of this study was to quantify the spatial variability of Rs over the growing season in a boreal aspen (*Populus tremuloides* Michx.) fire chronosequence. The chronosequence included three stands in northern Alberta; a post fire stand (1 year old, PF), a stand at canopy closure (9 years old, CC), and a mature stand (72 years old, MA). Soil respiration, temperature and moisture were measured monthly from May to August using an intensive spatial sampling protocol (n = 42, minimum lag = 2 m). Key aboveground and belowground properties were measured one time at each sampling point. No spatial structure was detected in Rs of the PF stand during the peak growing season (June and July), whereas Rs was auto-correlated at a scale of < 6 m in the CC and MA stands. The PF stand had the lowest mean Rs (4.60 μmol C m^-2^ s^-1^) followed by the CC (5.41 μmol C m^-2^ s^-1^), and the MA (7.32 μmol C m^-2^ s^-1^) stand. Forest floor depth was the only aboveground factor that influenced the spatial pattern of Rs in all three stands and was strongest in the PF stand. Enzyme activity and fine root biomass, on the other hand, were the significant belowground factors driving the spatial pattern of Rs in the CC and MA stands. Persistent joint aboveground and belowground control on Rs in the CC and MA stands indicates a tight spatial coupling, which was not observed in the PF stand. Overall, the current study suggests that fire in the boreal aspen ecosystem alters the spatial structure of Rs and that fine scale heterogeneity develops quickly as stands reach the canopy closure phase (<10 years).

## Introduction

The boreal ecosystem is the largest terrestrial biome on earth, representing about 25% of the global forested area and accounting for 289 Pg of carbon (C) most of which is in the soil [1–3]. Wildfire is one of the main drivers of C exchange in these ecosystems. Boreal forests are fire adapted and periodic wildfire causes stand renewal, landscape patchiness, large efflux of C to the atmosphere, large influx of stable C (in the form of black C) to the soil, and modifies the nutrient biogeochemical cycles [4–8]. CO_2_ efflux from soil or soil respiration (Rs) is one of the key ecosystem processes affected by wildfire. Rs is mainly composed of CO_2_ efflux from two different sources, viz., (i) autotrophic respiration (Ra), and (ii) heterotrophic respiration (Rh). Ra is from plant roots, rhizosphere mycorrhizae and living mosses, and Rh is from microbial activity [9]. In-situ soil respiration has been shown to be effective for tracking ecosystem recovery after stand replacing disturbance such as wildfire [10].

Significant efforts have been devoted to understand the potential sources of variation in Rs in different ecosystems [7, 11–13]. The two main state factors of variation thought to be responsible are soil temperature and moisture [14–16]. However, other soil properties such as organic matter quality, concentration of photosynthates in roots, and enzyme activity are also responsible for some of the variation in Rs [17, 18]. Despite technological progress in measuring Rs, there are few studies that have actually looked at the spatial variation in Rs as a means of quantifying these other potential sources of variation. Current prediction models are mainly based on the empirical relationship between Rs and soil temperature, which does not reflect the importance of other biotic and abiotic factors driving spatial variability in Rs, and therefore limits the mechanistic understanding of the variation in carbon efflux from belowground ecosystem [7].

In boreal ecosystem the spatial variation in Rs is mostly attributed to forest floor depth (FD) [2, 13, 19], substrate quality [2], root biomass [12, 20], vegetation type [2, 21], and soil temperature and moisture [22–26]. Spatial variability in Rs, however, depends on host of other abiotic and biotic factors such as texture and pore distribution in soil [27], stand structure [21, 28], nutrient availability [29], microbial dynamics [9, 30–32], and enzyme kinetics [33, 34] which all have spatial structure, making space an important component of variability in Rs [35, 36]. Therefore, spatial residuals can be used as a surrogate of unmeasured variables which will enable us to focus more directly on causal relationship between Rs and other factors [37]. Moreover, there is a general trend in the spatial Rs studies [2, 28, 38–41]to use either coefficient of variation as a measure of spatial variation, or non-spatial least square regression approach for modeling the drivers of Rs, both of which are unable to provide information on spatial extent (i.e. scale and magnitude) at which driving processes are functioning to create patterns in Rs, and prone to give erroneous parameter fit if data is spatially auto-correlated [42–44]. Detailed spatial characterization of aforementioned factors driving Rs is also necessary for predicting CO_2_ efflux at larger scale. This is especially important for boreal ecosystem where accumulation and turnover rate of soil C is highly variable [45, 46].

Variability in CO_2_ efflux from soil is generally high even within pure stands [21]. Therefore, it is not possible to generalize mechanisms of spatial heterogeneity in Rs across ecosystems having different stand characteristics. The boreal Rs literature [12, 19, 36, 47–51] are heavily focused on CO_2_ efflux from coniferous stands, specially black spruce (*Picea mariana*), and spatial processes creating Rs heterogeneity in these stands may not be fully applicable to the stands dominated by broadleaf species (e.g. aspen).

Wildfire is the main natural disturbance in the boreal ecosystems, and as important as decomposition in cycling soil C to the atmosphere [7]. Given the importance of wildfire in boreal C balance, its effects on the spatial distribution of Rs have not also been studied adequately, which is limiting our understanding of CO_2_ efflux in these ecosystems [48]. Wildfire reorganizes the spatial structure in soil processes by removing above ground organisms and consuming the organic layers [48, 52]. Stand-replacing fire can decrease spatial variability by homogenizing abiotic environmental conditions such as temperature and moisture. However, other factors such as organic matter quality, FD, and vegetation regrowth can also exert significant control where temperature and moisture are not limiting [7, 36]. We hypothesized that post fire Rs would have low heterogeneity and large scale spatial patterns, but over time as stands mature and structural complexity is recreated, we expect an increase in heterogeneity and fine scale patterns (Hypothesis 1). We expected to see a seasonal effect, with higher summer spatial variability (i.e. fine scale patchiness) than in the spring. Summer is the main growing season with the highest temperatures and precipitation, which should stimulate the major autotrophic and heterotrophic drivers of Rs (Hypothesis 2) [10, 53]. Availability of moisture and C quality are two major drivers that we expected to have significant influence on post-fire Rs (Hypothesis 3). The PF stand should experience a moisture limitation due to higher temperatures caused by the charred forest floor and an open canopy structure [54], along with a high number of aspen suckers and therefore high evapotranspiration. Carbon quality in the PF stand should also influence Rs, and as fire generally decreases soluble C, which is the energy source for microbial activity [55], we assumed that belowground controls would be stronger than aboveground controls in the PF stand (Hypothesis 4) as the old aboveground structure (e.g. tree canopies and living stems) was totally consumed by fire and the young regeneration might not have developed a strong enough effect on Rs to be detectable at this early stage of disturbance recovery [7, 54, 56, 57].

Understanding the mechanisms regulating the development of spatial heterogeneity in Rs with recovery from fire in the boreal aspen ecosystem is important. Besides deepening our ecological understanding, this study can also be used as a benchmark for measuring reclamation success of similar areas disturbed by industrial activity in the region. Data from this research will also be applicable for modeling C cycles in similar boreal ecosystems, as well as for the study design of any future soil respiration studies in the area. The specific objectives of this study were: i) to quantify the spatial variation in Rs along a chronosequence of stand development, iii) to characterize the seasonal pattern of spatial variation in Rs, and iii) to determine the principal factors controlling the spatial variation in Rs.

## Methods

### Study sites

The study was conducted in the Athabasca oil sand region (AOSR) around Fort McMurray, Alberta, Canada (56° 43′ N 111° 21′ W). The mean annual temperature in this region is 0.9°C and growing season (May–September) temperature is 13.3°C. Mean annual precipitation is 418.6 mm, of which 283.4 mm is rain during the growing season [58]. Soils in the study area are predominantly Orthic Gray Luvisol, sandy loam to silty loam, moderately well drained, and developed from till and glaciolacustrine sediments [59]. Three boreal aspen stands were used to create the studied fire chronosequence, a one year post fire (PF) stand, a 9 year old stand at canopy closure (CC), and a 72 year old mature stand (MA). The PF stand was created after the massive stand replacing fire (Richardson Fire; [60]) in 2011. Aspen was the dominant tree species and represented more than 95% of the basal area in all three sites. The maximum distance between sites was 34 km. The number of aspen suckers in the PF stand was counted as 230, 000–270, 000 stems/ha. The CC stand had a tree density of 1900 stems/ha and was different from the other sites in that it had a large amount of coarse woody debris (CWD) on the ground due to deadfall. The density in the MA stand was approximately 2150 stems/ha. According to the ecosite classification of northern Alberta, all the three sites fall under the d1 ecosite phase (low-bush cranberry Aw) [61]. Geographic location, fire history and dominant shrub, forb, grass and moss species of the study sites are given in Table A in S1 File.

### Soil Respiration measurements

**Fig 1:**
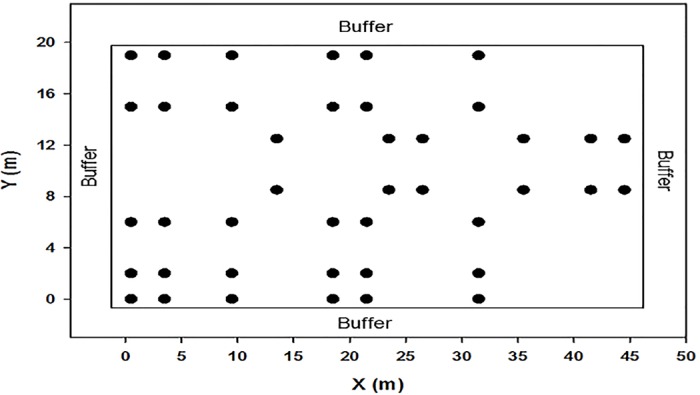
Lay-out of the spatial sampling protocol used to measure soil respiration (Rs) and the aboveground and belowground properties in the boreal aspen stands in northern Alberta along a fire chronosequence.

Sampling and *in-situ* measurements were carried out in a 50 m × 20 m plot at all the three sites. Plots were selected based on topography, slope, and aspect to make them comparable across the sites. A cyclic spatial sampling protocol with variable intervals [62] was used to capture both the scale and directionality in measured properties (Fig 1). In total 42 sampling points were established along 7 transects, within a 1000 m^2^ plot, which ensured a minimum detectable spatial lag of 2.0 m (Fig 1). Intervals between sampling points within each transect were 3, 6 and 9 m, and inter-transect intervals were 2 m and 4 m. The sampling orientation was reversed in the middle two transects to capture anisotropy. Respiration collars made of high grade PVC pipe (10 cm internal diameter) were installed at each sampling point. Collars were inserted 8 cm deep into the soil leaving 2 cm above the soil surface to house the respiration chamber. Side walls of the collars were drilled (3 to 5 holes) to allow lateral water movement. Collars were installed 48 hours prior to respiration measurements to avoid the initial flush of CO_2_ due to ground disturbance. CO_2_ concentration was measured using a portable dynamic closed chamber infrared gas analyzer (CIRAS 1) with a SRC-1 soil respiration chamber (PP systems, Hitchin Herts, UK). Efflux measurement criteria were set at 120 seconds (for the length of measurement) and 90 ppm (for the difference in CO_2_ concentration). Rs was measured monthly from June to August, 2012, and May 2013. Soil temperature and volumetric moisture content were also measured at a depth of 8 cm at each sampling point during the respiration measurements using soil thermometer and Theta probe and HH2 moisture meter (Delta-T, UK). Air temperature was also recorded every 30 minutes during the whole measurement period. All measurements occurred between 8:30 am to 1:30 pm to reduce the diurnal variation in soil respiration.

### Lab analyses

Soil samples (n = 42 per site) were collected at each spatial sampling point in August 2012 and included the entire FD and 5 cm of mineral soil. Samples were kept chilled with ice packs in the field and then stored at 4°C in the lab until further processing. After carefully removing the coarse roots and coarse fragments, samples (forest floor plus mineral soil) were homogenized. A sub-set of the samples were frozen at -20°C for analysis of extracellular enzyme activity. Approximately 75 to 100 g soil was incubated for 10 days at 25°C in sealed Mason jar with alkali trap (0.5 M NaOH) and basal respiration was calculated after titrating with 0.5 M HCl [63]. Microbial biomass C (MBC) and N (MBN) were measured on incubated samples using the fumigation extraction method [64]. Two sets of approximately 25 g of soil were extracted in 0.5 M K_2_SO_4_ (1:2 ratio), one after exposing to chloroform (CHCl_3_) for 96 hours. Dissolved organic C (DOC) and N (DON) were measured on both fractions using Shimadzu TOC-V/TN analyzer (Shimadzu Corp., Kyoto, Japan), and microbial biomass was determined by taking the difference of the two and no correction factor was used. Total C and total N were measured on oven dried (105°C) samples using a Costech 4010 Elemental Analyzer System (Costech Analytical Technologies Inc., Valencia, CA, USA).

Three enzymes were examined in this study including: (i) β-glucosidase (EC 3.2.1.21)which is responsible for breaking labile cellulose and other carbohydrate polymer chains, (ii) phenol oxidase (EC 1.10.3.2), which mainly degrades lignin and laccases, and (iii) peroxidase (EC 1.11.1.7) which also degrades lignin and polysachharide but uses H_2_O_2_ or secondary oxidants as electron acceptor [65]. β-glucosidase activity was measured using 4-methylumbelliferyl (MUB)-β -D-glucopyranosidase as a fluorimetric substrate, while the phenol oxidase and peroxidase activity were measured using 3,4-dihydroxy-L-phenylalanine as a colorimetric substrate [66, 67]. Assay and control wells were replicated 8 times. Activity rates (μmol of converted substrate g^-1^ soil hour^-1^) were calculated on an oven dry mass basis.

### Measurement of stand attributes

Stand characteristics were measured around each spatial sampling point (n = 42 per site). CWD cover was measured and number of aspen seedlings was counted within a 0.25 m^2^ frame in the PF stand, and within a 1 m^2^ frame in the CC and MA stands. Location (XY coordinates) of large trees was also measured in the PF and MA stands using a Nikon total station (Nikon DTM 352). Most of the standing dead trees were grounded in the CC stand and not considered for stem mapping. Canopy cover estimation was done using a convex densitometer and FD was measured using a ruler (average of three measurements). Fine root biomass (< 5 mm) (FRB) was estimated using the allometric equation developed in [68] (see S1 File). FRB value for each spatial point was taken from the estimated root biomass of the nearest tree (PF and MA stands) or saplings in the spatial grid (CC stand).

### Geo-statistics and other statistical analyses

Isotropic semi-variograms of the measured variables were calculated to examine the spatial autocorrelation in Rs and other attributes [69]. Data were log transformed prior to analyzing semi-variograms and variogram models. Five variogram models (Linear, Gaussian, Exponential, Spherical and Nugget) were tested to fit the empirical data. A combination of highest coefficient of determination (R^2^) and the lowest residual sum of square error was used to select the final model. Spatial dependence was calculated using the nugget coefficient, n_c_ which is a ratio of total variance (c_0_ + c_1_) and nugget variance (c_0_). The nugget variance represents the uncertainty caused by small-scale variation or sampling and measurement errors [70]. A nugget coefficient > 75 indicates strong spatial dependence, 25–75 indicates moderate dependence, and < 25 indicates poor or no spatial dependence [71]. Coefficient of variation (CV) was used to measureglobal variation and was calculated by dividing the standard deviation of Rs, soil temperature, and soil moisture by their means, and reported as a percentage. Cross-variograms were calculated for Rs and the aboveground and belowground variables to examine the scale of spatial relationships. A positive cross-variance indicates spatial association, whereas a negative variance means spatial dissociation. Fitted semi-variogram models were used for creating ordinary krieged maps of Rs. Variogram modeling and krieging interpolation was done using GS+ geostatistic software (V9.0, Gammadesign software). Further details on the semi-variogram models are given in S1 File.

Factors driving Rs and their seasonal influence in different stands were tested using spatial autoregressive (SAR) models. Both the spatial error (SAR_err_) and spatial lag (SAR_lag_) models were tested, and the one with lowest AIC and highest R^2^_adj_ (adjusted for number of predictors in the model) was selected [72, 73]. SAR analysis was done in R (R development Core Team, 2013) and Geoda, an open source geospatial software [74]. Details on SAR calculation and interpretations are given in S1 File.

Finally, oneway-ANOVA was used to differentiate between soil respirations of different sites in different seasons (SPSS Inc. Chicago, IL, USA). Multiple comparisons were made using Tukey HSD post hoc test (α = 0.05). Assumptions of normality and homoscedasticity were tested and transformations were made where necessary [18].

## Results

### Soil respiration (Rs) and aboveground and belowground factors

Soil respiration varied among stands (*p* < 0.01) and seasons (*p* < 0.000). Post fire stand had the lowest average growing season Rs (4.60 ± 0.17 μmol C m^-2^ s^-1^) followed by the CC (5.41 ± 0.28 μmol C m^-2^ s^-1^) and MA stand (7.32 ± 0.29 μmol C m^-2^ s^-1^). Lowest early summer (May) Rs was found in the CC stand whereas lowest late summer (June–August) Rs was in the PF stand (Table 1). With the measured rate, annual daytime C emission from the forest soil of MA stand during growing season (May–August) was 11.2 Mg C ha^-1^. In the PF and CC stands these values were 7.1 and 8.3 Mg C ha^-1^, respectively. Significant differences were also found in soil temperature and moisture in different stands (Table 2). A trend of increasing soil temperature was observed in all stands from May to July, but then dropped by August. Soil temperature ranged from 11.4 to 16.1°C in the PF stand, 6.9 to 16.1° in the CC stand, and 7.5 to 13.8°C in the MA stand. A trend of decreasing soil moisture was observed in all the stands except July in the PF stand.

**Table 1:** Seasonal mean, coefficient of variation (CV) and variogram parameters of soil respiration (μmol C m^-2^ s^-1^) measured in three boreal aspen stands in northern Alberta along a fire chronosequence. Different letters for the same month in each site indicate significant (*p* < 0.05) difference among sites.

Site	Month	Mean ^‡^Rs (± SE)	CV (%)	Range (m)	Spatial dependence	Dependence class	Model	R^2^
^**‡**^**PF**	**May**	3.16 (0.15)^A^	30.4	5.8	0.86	Strong	Spherical	0.34
	**June**	6.29 (0.38)^A^	39.4	> 23	0.53	Moderate	Exponential	0.45
	**July**	5.38 (0.30)^A^	36.5	^‡^ND	-	-	-	-
	**August**	3.57 (0.20)^A^	35.4	5.2	0.98	Strong	Spherical	0.21
^**‡**^**CC**	**May**	1.82 (0.14)^B^	48.3	4.4	0.97	Strong	Spherical	0.28
	**June**	4.27 (0.31)^B^	47	5.6	0.96	Strong	Spherical	0.41
	**July**	7.24 (0.55)^B^	48.6	4.8	0.84	Strong	Spherical	0.26
	**August**	8.30 (0.46)^B^	35.8	ND	-	-	-	-
^**‡**^**MA**	**May**	3.24 (0.17)^A^	34.2	8.0	0.95	Strong	Exponential	0.21
	**June**	9.23 (0.51)^C^	35.6	4.0	0.94	Strong	Gaussian	0.58
	**July**	9.40 (0.48)^C^	32.8	3.6	0.98	Strong	Spherical	0.28
	**August**	7.39 (0.46)^C^	40.1	5.0	0.99	Strong	Gaussian	0.78

^‡^PF = Post fire; CC = Canopy closure; MA = Mature stand; Rs = Soil respiration; ND = not detected

**Table 2:** Seasonal mean, coefficient of variation (CV), and variogram parameters of soil temperature (°C) and soil moisture content (m^3^ m^-3^) measured in three boreal aspen stands in northern Alberta along a fire chronosequence. Different letters for the same month in each site indicate significant (*p* < 0.05) difference among sites.

Site	Month	Factors	Mean (± SE)	CV (%)	Range (m)	Spatial dependence	Dependence class	Model	R^2^
^**‡**^**PF**	**May**	^**‡**^**ST**	11.38 (0.11)^A^	6.4	ND	-	-	-	-
		^**‡**^**SM**	0.22 (0.01)^B^	26.5	ND	-	-	-	-
	**June**	**ST**	12.2 (0.14)^A^	7.5	19	0.52	Moderate	Gaussian	0.33
		**SM**	0.20 (0.01)^B^	23	ND	-	-	-	-
	**July**	**ST**	16.1 (0.11)^B^	4.4	> 23	0.50	Moderate	Linear	0.24
		**SM**	0.22 (0.01)^A^	19	4.8	0.96	Strong	Spherical	0.22
	**August**	**ST**	15.2 (0.07)^A^	2.9	7.6	0.86	Strong	Gaussian	0.86
		**SM**	0.14 (0.003)^B^	18.5	ND	-	-	-	-
^**‡**^**CC**	**May**	**ST**	6.9 (0.31)^B^	28.9	6.4	0.99	Strong	Gaussian	0.82
		**SM**	0.23 (0.01)^AB^	24.7	> 23	0.71	Moderate	Gaussian	0.73
	**June**	**ST**	11.4 (0.17)^B^	9.5	> 23	0.85	Strong	Spherical	0.94
		**SM**	0.21 (0.01)^AB^	16.2	20	0.50	Moderate	Gaussian	0.47
	**July**	**ST**	16.1 (0.12)^B^	4.8	> 23	0.80	Strong	Gaussian	0.96
		**SM**	0.15 (0.01)^B^	26.6	> 23	0.63	Moderate	Gaussian	0.56
	**August**	**ST**	15.1 (0.10)^B^	4.4	> 23	0.99	Strong	Spherical	0.89
		**SM**	0.15 (0.004)^B^	20.5	3.6	0.99	Strong	Gaussian	0.42
^**‡**^**MA**	**May**	**ST**	7.49 (0.18)^B^	15.2	ND	-	-	-	-
		**SM**	0.25 (0.01)^A^	27.5	> 23	0.53	Moderate	Linear	0.52
	**June**	**ST**	^C^9.8 (0.08)^C^	5.4	> 23	0.67	Moderate	Spherical	0.76
		**SM**	0.22 (0.01)^A^	27.2	13.1	0.59	Moderate	Spherical	0.50
	**July**	**ST**	13.8 (0.06)^A^	3.1	6.4	0.91	Strong	Gaussian	0.52
		**SM**	0.15 (0.004)^BC^	21.7	ND	-	-	-	-
	**August**	**ST**	13.7 (0.06)^A^	2.8	6.5	0.89	Strong	Exponential	0.19
		**SM**	0.09 (0.003)^A^	25.3	4.2	0.99	Strong	Gaussian	0.56

^‡^PF = Post fire; CC = Canopy closure; MA = Mature stand; ST = Soil temperature; SM = Soil moisture

The aboveground and belowground properties also varied (*p* < 0.10) among the stands (Table B in S1 File). The MA stand had the highest MBC, MBN, DOC, Phenol oxidase, Total C, Total N, FRB, FD, and canopy cover. DOC increased with stand age, but not significantly, while DON was significantly higher in the PF stand than the CC and MA stands. BR was higher in the PF stand than the CC stand, but not the MA stand (Table B in S1 File).

### Spatial variation in Rs, soil temperature and moisture

Strong to moderate spatial dependency was observed in Rs, soil temperature, soil moisture, as well as other above and belowground properties in all three aspen stands (Table 1). Global variation (CV) in Rs did not follow any specific trend, but generally showed higher value during summer measurements (June and July). Overall, the CC stand had the highest global variation in Rs (35.8–48.6%) (Table 1). A gradual decrease in the global variability of soil temperature was observed in all stands from May to August. The global variability of soil moisture also decreased from May to July in the PF and MA stands and then increased, while no specific trend was found in the CC stand.

**Fig 2:**
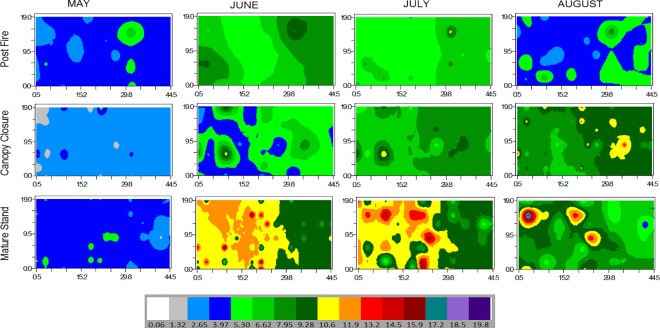
Krieged map of soil respiration (μmol C m^-2^ s^-1^) showing changes in different growing season months along a fire chronosequence of boreal aspen stands in northern Alberta

Soil respiration in the PF stand had a spatial range of 6 m in May and August, but very coarse range (> 23 m) in June and July. The spatial range of Rs in the CC stand varied between 4.4 m (May) to 5.6 m (June) to 4.8 m (July), but no spatial autocorrelation was detected in August (Table 1). Soil respiration in the MA stand had a spatial range ≤ 5 m throughout the growing season except in May (8 m). Seasonal changes in Rs in different stands are also shown in interpolated krieged maps which again are indicating an increase in patchiness with time since last disturbance (Fig 2).

Large scale spatial autocorrelation (≥ 19 m) was detected for soil temperature in the PF and CC stands during most of the growing season, and soil moisture did not show any detectable spatial autocorrelation except in July in the PF stand (4.8 m) and in August in the CC stand (3.6 m) (Table 2). The MA stand had large scale spatial range (> 23 m) in soil temperature and soil moisture in the early growing season which gradually became finer by August (4.2 m).

**Fig 3:**
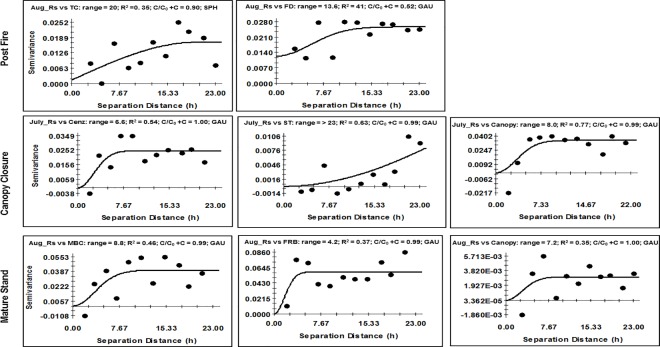
Cross-variograms between soil respiration (Rs) and the aboveground and belowground properties in the boreal aspen stands in northern Alberta along a fire chronosequence. (TC = Total organic C; MBC = Microbial Biomass C; Cenz = C mineralizing enzymes; FD = Forest floor depth; Tdist. = Distance to nearest tree; ST = Soil temperature; Canopy = Canopy cover; FRB = Fine Root Biomass; SPH = Spherical; GAU = Gaussian).

Cross-variogram analysis between Rs and aboveground and belowground variables indicated some spatial association at ranges smaller than the study area for all stands (Fig 3). For example, Rs in the PF stand was spatially associated with total C at 20 m and with FD at 13.6 m, whereas the CC stand was spatially associated with C enzymes at 7 m and canopy cover at 8 m. Likewise, Rs in the MA stand was spatially associated with MBC at 9 m, FRB at 4.2 m, and with canopy cover at 7 m.

### Spatial regressions

Spatial regression models indicated significant spatial and non-spatial control on Rs in all three stands along the fire chronosequence (Table 3). These models explained 18 to 52% of the variation in Rs in all stands. FD and soil moisture were the two most significant predictors of Rs in the PF stand, except in late summer (July) when DOC and DON were the main controlling factors. Results indicated some aboveground dependency in the CC stand, with FD and aspen sapling density consistently exerting positive control on Rs during the summer season (May to July; Table 3). However, the strongest control came from belowground properties such as enzyme activity and DOC, especially during June to August (Table 3). Soil MBC consistently had significant positive control and enzyme activity had mostly negative control on Rs in both PF and CC stands. Spatial relationship between Rs and above and belowground controls were more complex in the MA stand. The most significant aboveground control was found during July and August through the effect of FD, canopy cover, and tree distance, and for the latter two this got stronger in August. Among the belowground controlling factors, FRB appeared to be the most significant driver of Rs throughout the growing season.

**Table 3:** Spatial regression models and parameters of the seasonal relationships between soil respiration (Rs) and aboveground and belowground properties in three boreal aspen stands in northern Alberta along a fire chronosequence.

	Spatial regression models		*Lag coeff*. *(ρ)*	*Error coeff*. *(λ)*	*Log Likelihood*	*F*	*p*	*AIC*	*R*^*2*^_*adj*_
^‡^**PF**	**May**	Rs = ^**§§**^**0.44** + ^**§§**^0.07* **FD**– ^**†**^0.003*CWD– 0.002*BR– ^**††**^0.92*SM	-	^**†**^**-0.58**	39.71	5.42_5,33_	0.001	-69.43	0.34
	**June**	Rs = ^**§§**^**1.33** + ^**§§**^0.16*FD– ^**†**^0.01*CWD– ^**†**^0.07*FRB– ^**†**^0.003*DOC– ^**§**^0.06*Perox– ^**†**^2.53*SM	-	-0.04	1.05	3.78_7,32_	0.005	11.88	0.30
	**July**	Rs = ^**†§**^**1.46** + ^**††**^0.001*DOC + 0.004*BR– ^**††**^0.007*DON– ^**†**^0.05*ST	-	0.16	34.11	5.80_5,31_	0.001	-58.23	0.34
	**Aug**	logRs = 0.02 + ^**††**^0.28*logFD– ^**§**^0.02*logCWD– ^**§**^0.28*logPhenol	-	-0.08	71.24	4.26_4,37_	0.01	-134.49	0.18
^‡^**CC**	**May**	Rs = ^**†**^**0.24 +** ^**††**^0.02*FD + ^**†**^0.01*Sapling density– ^**§**^0.23*Bglu– ^**†**^0.23*Phenol– ^**†**^0.02* **MBCN +** ^**†**^0.01*ST	-	0.07	-54.44	3.99_7,30_	< 0.001	-94.88	0.31
	**June**	Rs = 0.26 + ^**§**^0.03*FD + ^**††**^0.04*Sapling density– ^**††**^1.29*Bglu + 0.05*Perox **+** ^**†§**^0.002*DOC– ^**†**^1.68*SM	-	^**†**^**0.30**	-10.47	3.46_7,32_	< 0.001	-6.95	0.33
	**July**	Rs = ^**††**^**-0.38 +** ^**†**^0.07*FD + ^**†**^0.04*Sapling density + ^**††**^0.14*Perox + 1.62*TN + ^**†**^0.21*ST	-	0.18	-8.85	5.73_6,31_	< 0.001	29.7	0.40
	**Aug**	Rs = ^**§§**^**1.60 +** ^**†**^0.09*FD + ^**††**^0.003*DOC– ^**†§**^7.16*SM	-	0.15	-16.52	8.13_4,37_	< 0.001	41.04	0.36
^‡^**MA**	**May**	Rs = ^**†**^**0.39** + ^**§§**^0.006*MBN + ^**§**^0.03*Perox + ^**§**^0.002*DOC– ^**†**^0.02*DON– ^**†**^0.05*TC -^**†**^0.58*SM	-	0.15	24.94	3.91_7,34_	< 0.005	24.94	0.29
	**June**	Rs = 0.25 + 0.04*FD + ^**§§**^0.09*FRB + ^**†**^0.003*DOC + ^**†**^2.7*Bglu + ^**†**^0.14*Phenol– ^**§**^1.60*SM	-	-0.01	-12.08	6.24_7, 32_	0.0001	38.18	0.43
	**July**	Rs = 0.12 + ^**††**^0.08*FD– ^**†**^0.18*Tdist + ^**§§**^0.10*FRB + ^**††**^0.001*MBC + ^**§**^2.28*Bglu + 0.10*Phenol– 0.01*BR	-	- 0.18	-14.29	4.75_8,33_	0.0008	44.58	0.40
	**Aug**	Rs = -2.68 + ^**††**^0.025*Canopy + ^**§**^0.03*FD– ^**†§**^0.16*Tdist + ^**§§**^0.08*FRB + ^**†§**^0.001*MBC + ^**††**^0.09*Perox– ^**§**^0.07*TC	^**††**^**0.29**	-	3.34	5.96_8,32_	0.0001	11.31	0.52

§ *p* ≤ 0.10

† *p* < 0.05

†† *p* < 0.005

†§ *p* < 0.0005

§§ *p* < 0.00005

^‡^PF = Post fire; CC = Canopy closure; MA = Mature stand; MBC = Microbial biomass C (μg g^-1^ soil); MBN = Microbial biomass N (μg g^-1^ soil); MBCN = Microbial C to N ratio; BR = Basal respiration (μg CO_2_-C g^-1^ soil day^-1^); DOC = Dissolved organic C (μg g^-1^ soil); DON = Dissolved organic N (μg g^-1^ soil); Bglu = β-1,4 glucosidease (nmol g^-1^ soil hour^-1^); Phenol = Phenol Oxidase (nmol g^-1^ soil hour^-1^); Perox = Peroxidase (nmol g^-1^ soil hour^-1^); TC = Total C (%); TN = Total N (%); FD = Forest floor depth (cm); FRB = Fine root biomass (kg stem^-1^; g stem^-1^ in CC); Tdist = Distance to nearest tree (cm); CWD = Coarse woody debris cover (%).

## Discussion

The rate of Rs found here corroborates previous studies done in boreal aspen forests [25, 53, 75]. Russell and Voroney [25] reported an efflux of 2.27 μmol C m^-2^ s^-1^ in May and maximum 9.09 μmol C m^-2^ s^-1^ in July in a 70 years old boreal aspen forest. The corresponding values in our study were 3.24 and 9.40 in May and July, respectively. The lower Rs in July in the PF stand can be attributed to the decrease in autotrophic respiration due to the premature defoliation of aspen seedlings in July caused by ink spot disease, a common feature in young aspen stands regenerating from suckers [76].

Mean Rs in the PF stand was 15% and 37% lower than the CC and MA stands, respectively. Studies performed in pyrogenic ecosystems often report similar effects of fire on soil respiration [77]. Fire can have direct negative effects on Rs in a number of ways including removal of the organic layer, reduction in microbial biomass, and thermal conversion of C to more recalcitrant forms [78]. The decrease in Rs in the PF stands can be attributed to a decrease in autotrophic respiration, however other factors such as soil moisture and microbial activity have also been found to be important in some studies [79, 80]. Our results confirm the importance of both above and belowground factors for determining mechanistic control on Rs and its spatio-temporal variability.

### Spatial patterns

The current study showed that spatial heterogeneity in Rs increased with increasing stand structural complexity and age in boreal aspen stands (Table 1 and Fig 2). Soil respiration in the CC and MA stands had stronger spatially predictable heterogeneity than in the PF stand, which appeared to be more random. These findings support our first hypothesis that fine scale heterogeneity in Rs would develop overtime.

Our results and the literature suggest that the lack of spatial structure in Rs post-fire can be attributed to lower variability in FD, an open canopy structure, and lower living FRB [2, 12, 13]. Although mid growing season Rs had large scale spatial structure in the PF stand, it exhibited fine scale structure in the early and late growing season. At these times, aspen growth was probably limited by environmental and physiological conditions such as low soil temperature in May and defoliation in August. This indicates that fire might have created patchiness in heterotrophic respiration, but homogenized the distribution of living FRB and therefore autotrophic respiration [51, 81]. Stand replacing fire usually consumes aboveground living biomass, which causes fine root mortality and decomposition, and recovery can take several years [82, 83].

The large scale or non-detectable spatial patterns in soil temperature and soil moisture in the PF stand was expected and is an example of the typical spatial structure of these variables in fire disturbed ecosystems with open canopies [84]. The detectable spatial patterns in these variables in the CC stand, and the medium spatial pattern (≤ 13 m) in the MA stand indicates recovery of spatial variability due to the development of above and belowground features such as forest floor, fine root development, and enzyme activity. Soil physical conditions in the post-fire environment may indirectly control Rs mainly through the fluctuations in soil temperature and moisture. However, such physical control remains significant only for a short period before vegetation control takes over [85, 86]. These findings again support our hypothesis that stand-replacing fire can decrease spatial variability in Rs by homogenization of abiotic environmental conditions such as temperature and moisture, and fine scale heterogeneity can develop over time as stands mature and structural complexity is recreated. The fine scale spatial autocorrelation of Rs in the CC and MA stands might have originated from the fine scale spatial association between stand (canopy cover, FD, and FRB) and soil microbial attributes (Table 1 and Fig 3). In a boreal mixedwood (aspen-black spruce) fire chronosequence, Lavoie and Mack [19] showed a gradual decrease in the scale of spatial variability in soil microbial properties and FD with time since last fire.

### Seasonal variation

Only the MA stand followed our hypothesized trend of fine scale structure in Rs and driving factors during the peak growing months (July and August) (Table 1). This indicates that not only the spatial structure of Rs was disturbed by wildfire, but that the seasonal pattern was also disrupted, and the recovery was not fully established even at the canopy closure phase. Forest structure in the CC stand was probably not complex enough to create the hypothesized seasonal trend in Rs. In the boreal ecosystem, post fire development of vertical structure including canopy transition and gap dynamics may take more than 25 to 35 years after stand initiation [87]. Moreover, the large amount of CWD in this stand might have masked the effect exerted by aboveground factors (e.g. canopy overlap, understory vegetation, and sapling density). Lee [88] found that all the standing snags had fallen down within 15 years of fire in a boreal aspen stand. This pulse of CWD after fire usually disappears 50–100 years [89, 90] as does its effect of Rs as evidenced by the MA stand.

### Spatial controls

The spatial pattern in residual error of the Rs regression models were considered in our study through the SAR approach, and therefore the resulting relationships between Rs and predictor variables can be treated as mechanistic [91]. Neither of the measured environmental factors (soil temperature and moisture) appeared as the strongest controlling factor of Rs in any of the stands. However, a significant negative effect of soil moisture was found during the early growing season months in all stands. Such a negative control of moisture could be the residual signature of snow melt. High moisture (> 20%) content had an overall negative relationship with Rs, but lower moisture contents did not follow any specific trend (data not shown). The expected positive control of DOC and soil moisture on Rs in the PF stand was only supported partially (in July) and the relationship appeared to be changing seasonally (negative in early and positive in late growing season) indicating a bimodal control on Rs. The CC stand, however, showed such evidence of positive control from DOC. The evaporative demand for moisture from the growing vegetation in the PF stand might have increased during the late growing season and created a moisture limited condition which in turn generated a positive feedback between soil moisture and Rs. A high evaporative demand in post fire aspen stand is common and likely to negatively affect belowground C balance in fire prone boreal ecosystem [92]. The negative control of DOC and soil moisture on Rs in the PF during the early growing season can be attributed to the high moisture availability from the spring snow melt; not due to the lower DOC concentration in soil solution per se [93, 94]. Although a similar seasonal trend in the soil moisture and DOC in post fire stands has been reported by other studies [55, 95, 96], their cumulative effect on the spatial variability in Rs seems unclear and require further investigation.

Some of the strongest controls on Rs were found from enzyme activity although the relationship was mostly negative in the PF and CC stands, and mostly positive in the MA stand. The negative enzymatic control on Rs might be due to the end product inhibition of C mineralizing enzymes. Presence of readily available C inhibits C mineralizing enzyme activity [97, 98]. Fire effects in broadleaf forest with low combustibility, as in the case of aspen, has been shown to be responsible for higher input of biodegradable C in soil [78].

FD had a significant positive effect on Rs which confirms similar findings from several other studies in boreal ecosystems [2, 12, 13]. The forest floor layer including the top 3–5 cm mineral soil is the most biotic active zone in the boreal ecosystems [99]. Most the of the FRB in boreal ecosystems is found in the organic layer and mineral soil interface, which makes the forest floor a very important regulator of the soil respiration [100]. Fire consumed a portion of the organic layer in the PF stand, but the residual organic layer became an important microbial hub and zone of root proliferation. This zone might have captured thermally altered C substrates [101] and this could have initiated the positive feedback to Rs.

A joint aboveground and belowground control on Rs was more evident in the CC and MA stands. FD, sapling density, and enzyme activity showed consistent positive control on Rs in the CC stand throughout the growing season which indicates recovery of spatial coupling in this stand. The MA stand, however, showed the strongest spatial coupling between aboveground-belowground variables and Rs (maximum R^2^ 0.52, Table 3). Evidence of such aboveground-belowground spatial coupling has been reported previously in pyrogenic ecosystems for other biogeochemical properties such as nutrient cycling [36], but findings from the current study suggest that ecosystem processes might share a common regulating mechanism as in the case of Rs; an amalgamative process which represents a cumulative effect of microbial, rhizosphere, and root respiration [10].

Soil respiration in the CC and MA stands also had significant negative controls from enzyme activity but only during the early growing season (May). This may suggest that Rs in the pyrogenic boreal ecosystem is not substrate limited in the early growing season, and the concentration of labile C is probably higher than the microbial demand. Significant positive relationship, however, in the later months, particularly in the CC and MA stands, indicates that there is a biotic demand for C during the peak growing season (June–August). The source of respiration in boreal ecosystems has been shown to be changing from stored C pool in the early growing season to photosynthetic products in peak growing season [7, 102]. Hogberg et al. [103] emphasized that photosynthates drive the peak to late growing season Rs in boreal ecosystem, and belowground C allocation needs to be considered more than the seasonality in determining the respiratory loss of C. Our findings corroborate these studies from a microbial perspective. Peroxidase enzyme is mainly responsible for degrading recalcitrant and aromatic C structure [104]. A gradual late emergence of peroxidase enzyme in the spatial Rs models is probably giving an indication that the shift in microbial foraging from labile to more recalcitrant organic matter depends on the supply of labile C [97], and in the MA stand it happens much later in the growing season than the young fire disturbed stands due to having a greater supply of labile C.

Significant space effect in the Rs of PF stand in May was unexpected, but not surprizing given that the some of the variables important for biogeochemical cycling in the post fire ecosystems such as charcoal and organic matter quality were not measured directly in our study. The distribution of charcoal can have a significant effect on the spatial variability of biotic properties through its high sorption capacity and porous structure [35, 105]. In pyrogenic boreal ecosystems, this might be important during snow melt and the subsequent flush of C and nutrients. Therefore, we assume that charcoal directly affected C mineralization through variable water retention in the forest floor. Despite having a complex spatial prediction model, a significant space factor was also detected in the lag term of the spatial model of August Rs in the MA stand. This indicates that a finer spatial lag would probably be more appropriate for quantifying Rs dependency in this stand.

## Conclusions

Studies on post-fire spatial heterogeneity in Rs in boreal ecosystems are few despite its importance in modeling and predicting future net C exchange. To our knowledge, this is the first study which simultaneously looked at the development of spatial heterogeneity in Rs, both seasonally and along a chronosequence of fire disturbance, and modelled the mechanistic relationships among the driving factors (abiotic, plants and microbes) after considering their spatial autocorrelations. Based on our findings, we conclude that stand-replacing fire has created large scale spatial pattern (less spatial variability) in the Rs of boreal aspen ecosystem, and a development of fine scale heterogeneity (more spatial variability) was found along the chronosequence. The similar spatial structure in Rs and its driving mechanisms in the CC and MA stands implies a quick recovery in the spatial heterogeneity of Rs within 9 years after fire disturbance.

A belowground microbial control on Rs in the PF spatial model suggests a dominance of heterotrophic contribution. However, the emergence of both autotrophic and heterotrophic factors in the spatial models of the CC and MA stands indicates an established aboveground-belowground feedback loop. FD could be used as a prime predictor of Rs in all seasons, however, variable combinations of FRB, enzyme activity, and environmental factors (temperature and moisture) would be required for seasonal prediction and process based modeling. The significant space term in the MA stand during the early fall indicates spatial mechanisms of Rs operates at much finer scale than the used 2 m scale in this study. The current study also supports the previously hypothesized trend of C balance in pyrogenic boreal ecosystem that fire reduces respiration and that post fire upland boreal ecosystems are probably a C sink rather than a source.

## Supporting Information

S1 File This file contains information on study sites, semi-variogram models, and spatial autoregression analysis used in the current study. **Table A:** Geographic locations, fire history and dominant understory vegetation in the study sites. **Table B:** Mean, coefficient of variation (CV), and range of aboveground and soil bio-chemical properties in three boreal aspen stands in northern Alberta along a fire chronosequence. Different letters for the same property indicates significant difference among sites (*p* < 0.10).
(DOCX)
