# Supplementary material for: Spatial Patterns of Soil Respiration Links Above and Belowground Processes along a Boreal Aspen Fire Chronosequence
Source: PLoS One. 2016 Nov 10;11(11):e0165602. doi: 10.1371/journal.pone.0165602 (PMC5104365; doi:10.1371/journal.pone.0165602)
Supplement: S1 File — Table A: Geographic locations, fire history and dominant understory vegetation in the study sites. Table B: Mean, coefficient of variation (CV), and range of aboveground and soil bio-chemical properties in three boreal aspen stands in northern Alberta along a fire chronosequence. Different letters for the same property indicates significant difference among sites (p < 0.10). (DOCX) [file pone.0165602.s001.docx]

**Supporting information file S1**

**Spatial Patterns of Soil Respiration Links Above and Belowground Processes along a Boreal Aspen Fire Chronosequence**

Sanatan Das Gupta^1*^ and M.Derek Mackenzie^2^

^1^Natural Resources Canada, Canadian Forest Service, Northern Forestry Centre, Edmonton, AB, Canada

^2^Department of Renewable Resources, University of Alberta, Edmonton AB, Canada

^*^Corresponding author

Email: [sanatan@ualberta.ca](mailto:sanatan@ualberta.ca)

**Semi-variogram**

Semi-variograms were calculated using the following equation:

$\gamma_{x}\left( h \right)=\frac{1}{2N(h)}\sum_{i=1}^{N(h)} {(X_{i}-X_{i+h})}^{2}$ (1)

where, γ(h) is semi-variance, h is separation distance, X is variable of interest, and N(h) is the total pair of sample points separated by distance h [1].

Five theoretical semi-variogram models were tested in this current experiment viz. Spherical, Exponential, Gaussian, Linear and Nugget models. These models can be classified according to their asymptotic behaviour such as (i) The Spherical, Exponential and Gaussian models are characterized by having a sill (the distance at which semi-variance is maximum) and a range (the distance after which semi-variance follows a random pattern), (ii) The linear model, where semi-variance sill is larger than the plot scale, and (iii) The nugget model, where no spatial autocorrelation is detected at a given scale. A 23 m lag distance was used for semi-variogram calculation in this study as this was the reliable limit for detecting spatial structure given that the maximum distance in the sampling protocol was 45 m.

Cross-variograms were used to detect the scale dependant spatial association between soil respiration and controlling variables. The Cross-variance between two scale dependent variables was calculated as below:

$\gamma_{xy}\left( h \right)= \frac{1}{2N(h)}\sum_{i=1}^{N(h)} (X_{i}-X_{i+1})(Y_{i}-Y_{i+h})$ (2)

where, $\gamma_{\mathrm{xy}}\left( h \right)$ is the experimental cross-semivariogram of n spatial observations of variable X and Y, N(h) is the number of observations separated by a distance h and X_i_ is the location, i = 1, ... n [1].

**Spatial regression model**

Spatial regression models were used to detect factors spatially controlling soil respiration in the stands recovering from fire disturbance and at maturity. Theoretically, the spatial autoregression (SAR) model adds a spatial autocorrelation term in the standard ordinary least square regression (OLSR) model, where contiguity of sampling location is defined by a spatial weight matrix [2]. SAR models commonly take two forms, spatial lag model (SARlag) where spatial autocorrelation term is associated with the response variables and the spatial error model (SARerr) which accounts for spatial trend by incorporating the autoregressive process in the error term. SARlag has the following form:

*Y = Xβ + ρWY + ε, ε ~ N(0, σ^2^)* (3)

Where X is independent variable, *β* is regression coefficient, *ρ* is the autoregression coefﬁcient, *WY* expresses the spatial autocorrelation in the response variable Y (spatially lagged dependent variable) and *ε* is the random error term.

SARerr model has the following form:

*Y = Xβ + ε; ε = λWε + ξ, ε ~ N(0, σ^2^)* (4)

where *ε* is the random error term where *λ* is the spatial autoregression coefﬁcient, *Wε* represents the spatial structure (W) in error term (*ε*) and *ξ* is the independent error term after accounting for spatial relationship.

Moran’s index was used to determine the spatial dependency of regression model residual. The equation was used for calculating Moran’s *I*:

$I=\frac{n}{\sum_{i=1}^{n} {(X_{i}-\bar{X})}^{2}}\frac{\sum_{i=1}^{n} \sum_{j=1}^{n} W_{ij}(X_{i}-\bar{X})(X_{j}-\bar{X})}{\sum_{i=1}^{n} \sum_{j=1}^{n} W_{ij}}$ (5)

where n is the number of observations, *X* is the model residual value for each sampling point, $\bar{X}$ is the mean of the residuals and *W_ij_* is the weight matrix. Distance based contiguity spatial weights matrix was used in this analysis. The threshold distance to avoid island formation was calculated by Euclidean distance between sampling points. The minimum threshold distance for the current sampling protocol was 4.0 m.

Best candidate spatial regression models were selected by stepwise selection using the “step” function in R [3]. Multicollinearity in the spatial regression models with lowest AIC were tested by variance inflation factor (VIF) using the “vif” function in the car package of R [4]. Variables with VIF more than 3 were removed from the model. Normality in the model residuals was tested by Jarqua-Bera test [4] and heteroscedasticity was tested by Breusch-Pagan and Koenker-Bassat test [5].

**Fine root biomass estimation**

Aspen fine root biomass (< 5 mm; FRB) in the PF and MA stand was estimated using the allometric equation developed by [6]. Fine root biomass value for each spatial point was taken from the estimated root biomass of the nearest tree (as close as 0.10 m).

The following equation was used to calculate the FRB:

Fine root biomass (kg) = (0.0113 × 2.0711*DBH) * 1.125 (6)

where, DBH = Diameter at breast height (1.37 m)

**Table A: Geographic locations, fire history and dominant understory vegetation in the study sites.**

| Site | GPS coordinates | Fire year | Common understory vegetation |
| --- | --- | --- | --- |
| Post Fire (PF) | 57° N 18′ 0.73′′ N  111° 40′ 49.28′′ W | 2011 | †Shrub – PR, LC, TF, GA, SA, HZ  Forb – SR, FW, BB, VT, NB, PC, HS, RB, WS, SS, PW, BC  Moss – Schreber’s moss |
| Canopy Closure (CC) | 57° 07′ 9.78′′N  111° 36′ 22.74′′ W | 2003 |  |
| Mature Stand (MA) | 57° 1′ 26.41′′N  111° 55′ 39.81′′ W | 1972 |  |

†PR = Prickly rose (*Rosa acicularis*); LC = Low-bush cranberry (*Viburnum edule*); TF = Twin flower (*Linnaea borealis*); GA = Green alder (*Alnus crispa*); SA = Saskatoon (*Amelanchier alnifolia*); HZ = Hazelnut (*Corylus cornuta*); SR = Sarsaparilla (*Aralia nudicaulis*); FW = Fireweed (*Epilobium angustifolium*); BB = Bunchberry (*Cornus canadensis*); VT = Vetchling (*Lathyrus ochroleucus*); NB = Northern bedstraw (*Galium boreale*); PC = Palmate leaved coltsfoot (*Petasites frigidus*); HS = Honeysuckle (*Lonicera dioica*); RB = Raspberry (*Rubus idaeus.*); WS = Wild strawberry (*Fragaria vesca*); SS = Solomon’s seal (*Maianthemum stellatum*); PW = Pink whitegreen - ; BC = Bishop’s cap (*Mitella nuda*); Schreber’s moss (*Pleurozium schreberi*).

**Table B: Mean, coefficient of variation (CV), and range of aboveground and soil bio-chemical properties in three boreal aspen stands in northern Alberta along a fire chronosequence. Different letters for the same property indicates significant difference among sites (*p* < 0.10).**

| **Factors** | **PF** | | | | **CC** | | | | | **MA** | | | | |
| --- | --- | --- | --- | --- | --- | --- | --- | --- | --- | --- | --- | --- | --- | --- |
|  | **Mean (**± **SE)** | **CV (%)** | **Min.** | **Max.** |  | **Mean (**± **SE)** | **CV (%)** | **Min.** | **Max.** |  | **Mean (**± **SE)** | **CV (%)** | **Min.** | **Max.** |
| **^†^MBC** | 189.94 (9.47)^B^ | 32.9 | 73.1 | 347.2 |  | 190.0 (11.7)^B^ | 40.2 | 68.1 | 404.7 |  | 362.0 (0.21)^A^ | 37.8 | 156.7 | 768.9 |
| **MBN** | 30.0 (1.56)^A^ | 33.8 | 10.4 | 57.0 |  | 23.8 (1.36)^B^ | 37.1 | 8.64 | 44.5 |  | 50.11 (2.62)^C^ | 33.9 | 26.8 | 97.4 |
| **DOC** | 86.8 (6.94)^A^ | 51.8 | 26.3 | 267.7 |  | 89.3 (8.04)^A^ | 58.3 | 35.2 | 311.0 |  | 97.4 (5.53)^A^ | 36.8 | 30.9 | 190.7 |
| **DON** | 17.2 (1.08)^A^ | 40.7 | 5.28 | 32.4 |  | 7.16 (0.75)^B^ | 67.9 | 2.57 | 30.2 |  | 9.57 (0.60)^C^ | 40.7 | 3.91 | 23.9 |
| **BR** | 34.83 (1.12)^AC^ | 20.9 | 17.8 | 50.4 |  | 22.21 (1.22)^B^ | 35.5 | 7.82 | 43.05 |  | 33.73 (1.04)^C^ | 20 | 21.2 | 49.5 |
| **Bglu** | 0.12 (0.01)^A^ | 34.3 | 0.06 | 0.25 |  | 0.11 (0.01)^AB^ | 65.3 | 0.03 | 0.35 |  | 0.09 (0.01)^B^ | 41.9 | 0.04 | 0.24 |
| **Perox** | 4.05 (0.17)^AB^ | 27.9 | 1.33 | 6.07 |  | 4.43 (0.16)^A^ | 23.6 | 2.16 | 6.60 |  | 3.71 (0.19)^B^ | 34.2 | 0.88 | 7.39 |
| **Phenol** | 0.39 (0.02)^A^ | 28.9 | 0.12 | 0.61 |  | 0.41 (0.01)^AB^ | 21.5 | 0.24 | 0.66 |  | 0.69 (0.13)^C^ | 123.1 | 0.09 | 4.10 |
| **Cenz** | 4.57 (0.18)^A^ | 25.2 | 1.86 | 6.59 |  | 4.95 (0.17)^A^ | 21.9 | 2.59 | 7.16 |  | 4.51 (0.26)^A^ | 37.9 | 1.32 | 11.6 |
| **TC** | 3.15 (0.14)^A^ | 28.2 | 1.24 | 4.87 |  | 3.51 (0.22)^AB^ | 40.6 | 1.05 | 7.78 |  | 4.10 (0.17)^C^ | 27.3 | 2.51 | 6.49 |
| **TN** | 0.14 (0.01)^A^ | 23.1 | 0.07 | 0.21 |  | 0.16 (0.01)^AB^ | 27.1 | 0.06 | 0.24 |  | 0.19 (0.01)^C^ | 26.4 | 0.11 | 0.32 |
| **TC:TN** | 22.85 (0.61)^A^ | 17.4 | 16.9 | 36.6 |  | 22.8 (1.12)^A^ | 31.8 | 16.3 | 47.7 |  | 21.65 (0.73)^A^ | 21.8 | 17.6 | 36.3 |
| **FRB** | 2.91 (0.31)^A^ | 69.7 | 0.66 | 7.95 |  | - | - | - | - |  | 4.20 (0.36)^B^ | 52.8 | 0.97 | 10.8 |
| **Sapling density** | - | - |  |  |  | 7.14 (0.44) | 40.6 | 1.5 | 14 |  | - | - | - | - |
| **FD** | 5.18 (0.17)^A^ | 21.9 | 2.5 | 8 |  | 5.16 (0.25)^A^ | 31.3 | 2 | 9 |  | 9.8 (0.28)^B^ | 18.8 | 6 | 14 |
| **Canopy** | - | - |  |  |  | 74.7 (2.79)^A^ | 24.2 | 31.4 | 96.8 |  | 93.5 (0.78)^B^ | 5.4 | 77.1 | 99.0 |
| **CWD** | 6.19 (1.18)^A^ | 124 | 0 | 35 |  | 24.4 (2.90)^B^ | 77.4 | 0 | 75 |  | 9.33 (1.45)^C^ | 101 | 0 | 35 |

^†^MBC = Microbial biomass C (µg g^-1^ soil); MBN = Microbial biomass N (µg g^-1^ soil); MBCN = Microbial C to N ratio; BR = Basal respiration (µg CO_2_-C g^-1^ soil day^-1^); DOC = Dissolved organic C (µg g^-1^ soil); DON = Dissolved organic N (µg g^-1^ soil); Bglu = β-1,4 glucosidease (nmol g^-1^ soil hour^-1^); Phenol = Phenol Oxidase (nmol g^-1^ soil hour^-1^); Perox = Peroxidase (nmol g^-1^ soil hour^-1^); TC = Total C (%); TN = Total N (%); FD = Forest floor depth (cm); FRB = Fine root biomass (kg stem^-1^; g stem^-1^ in CC); CWD = Coarse woody debris cover (%).

**References**

1. Yates S, Warrick A. Geostatistics. Methods of Soil Analysis: Part 4 Physical Methods: Soil Science Society of America; 2002. p. 81-118.

2. Anselin L. Spatial Econometrics. In: Baltagi BH, editor. A Companion to Theoretical Econometrics. MA, USA: Blackwell Publishing Ltd.; 2001. p. 310-330.

3. Venables WN, Ripley BD. Random and mixed effects. Modern Applied Statistics with S: Springer; 2002. p. 271-300.

4. Kutner MH, Nachtsheim CJ, Neter J, Li W. Applied linear statistical models: McGraw-Hill Irwin New York; 2005.

5. Anselin L. Exploring spatial data with GeoDaTM: a workbook. Urbana. 2004;51:61801.

6. Chen W, Zhang Q, Cihlar J, Bauhus J, Price DT. Estimating fine-root biomass and production of boreal and cool temperate forests using aboveground measurements: a new approach. Plant Soil. 2004;265(1-2):31-46.
